# Evaluation of the merit of the methanolic extract of *Andrographis paniculata* to supplement anti-snake venom in reversing secondary hemostatic abnormalities induced *by Naja naja* venom

**DOI:** 10.1007/s13205-021-02766-z

**Published:** 2021-04-21

**Authors:** Akshatha Ganesh Nayak, Nitesh Kumar, Smita Shenoy, Maya Roche

**Affiliations:** 1 Department of Biochemistry, Melaka Manipal Medical College (Manipal Campus), Manipal Academy of Higher Education, Manipal, Karnataka India; 2 Department of Pharmacology and Toxicology, National Institute of Pharmaceutical Education and Research (NIPER), Hajipur, Bihar India; 3 Department of Pharmacology, Kasturba Medical College, Manipal Academy of Higher Education, Manipal, Karnataka India

**Keywords:** Activated partial thromboplastin time, Hemostatic, Methanolic extract of *Andrographis paniculata*, *Naja naja*, Prothrombin time, Supplement, Thrombin time

## Abstract

Increasing evidence suggests a sizable involvement of hemotoxins in the morbidity associated with envenomation by the Indian spectacled cobra, *Naja naja* (N.N). This study investigates the ability of Indian polyvalent anti-snake venom (ASV), methanolic extract of *Andrographis paniculata* (MAP) and their combination in reversing the hemostatic abnormalities, viz. activated partial thromboplastin time(aPTT), prothrombin time(PT) and thrombin time(TT) in citrated plasma. These parameters were assessed in 2 groups of experiments. Group 1: Without the prior incubation of plasma with venom and Group 2: With prior incubation of plasma with venom for 90 min at 37°C. Venom caused significant (*p* < 0.001) prolongation in aPTT (175%), PT (49%) and TT (34%) in Group 1 and ASV could completely bring them back to normal. MAP showed a concentration-dependent reversal in aPTT, normalization of PT and prolongation of TT. When low concentration of ASV was supplemented with MAP, their combined effect in normalizing aPTT and PT improved by 37% and 26% respectively when compared to ASV alone. In Group 2, venom caused significant (*p* < 0.001) prolongation in aPTT (231%), PT (312%) and TT (245%). ASV had limited effect in reversing aPTT (52%), TT (31%) but completely normalized PT. MAP was marginally effective in reversing the prolonged aPTT and PT but caused further prolongation of TT. Combination of ASV and MAP was more effective than ASV alone in reversing venom-induced increase in aPTT (52%) and PT (29%). The study proved that, a drastic reduction of ASV by 70%, could be effectively supplemented by MAP in combating hemostatic abnormalities induced by NN venom.

## Introduction

The venom of the Indian spectacled cobra, *Naja naja* (N.N) is a cocktail of 81 different toxins (Choudhury et al., 2017). The venom is generally known to be neurotoxic due to the presence of multiple neurotoxins with different mechanisms of action (Paoli et al., 2009; Barber, Isbister and Hodgson, 2013; Ranawaka, Lalloo and de Silva, 2013; Urs et al., 2014). These cause systemic paralysis of muscles, including those involved in respiration, leading to death (Ahmed et al., 2008). Increasing evidence suggests a sizable involvement of hemotoxins in the morbidity associated with N.N bite, causing wide-spread changes in hemostasis. These effects are mediated by toxins that affect platelet aggregation (Kumar et al., 2010), coagulation (Berling and Isbister, 2015), hemolysis (Dissanayake et al., 2018) and fibrinogenolysis (Gowda et al., 2006; Kumar et al., 2010).

The venom of N.N is primarily anticoagulant. The 5′-nucleotidase and NnPLA_2_-I, a phospholipase with molecular weight of 15.2 KDa, are known to cause an anticoagulant effect (Dhananjaya et al., 2006; Dutta, Gogoi and Mukherjee, 2015). There are conflicting reports regarding the ability of the venom to prolong the prothrombin time (Gowda et al., 2006; Gowtham et al., 2012). The venom is also known to prolong the re-calcification time (Gowda et al., 2006), activated partial thromboplastin time (aPTT) and thrombin time (TT) (Gowtham et al., 2012). Though the venom promotes clot formation *in-vitro*, by activating prothrombin, the clots formed are abnormally low in elasticity due to decreased clot retraction (Sundell et al., 2003). N.N venom hydrolyses the α-chain of fibrinogen (Gowda et al., 2006) and shows feeble plasmin-like activity on fibrin clot (Gowtham et al., 2012). NN-PF3, a metalloprotease from N.N venom with molecular mass 67.81 KDa, prolonged aPTT, PT, TT, exhibited fibrin(ogen)olytic activity which was inhibited by polyvalent anti-snake venom (ASV). It did not affect the activity of thrombin. It inhibited platelet aggregation primarily by affecting α_2_β_1_ integrin (Kumar et al., 2011). Crude N.N venom and purified NND-IV-PLA_2_ from the venom, inhibited ADP and epinephrine-induced platelet aggregation (Gowtham et al., 2012, Satish et al., 2004). Widespread changes affecting different stages of clotting such as R- time, K-time, angle, amplitude and fibrin(ogen)olysis were well documented in real-time using thromboelastography (TEG). These changes were completely reversed by ASV and the methanolic extract of *Andrographis paniculata* (MAP) when used individually or in combination (Nayak, Ahammad, et al*.*, 2020).

In India, the only available antidote to envenomation with N.N is a polyvalent ASV which claims to neutralize the venoms of the four predominant snake species of the subcontinent, i.e., *Daboia russelli*, *Bungarus caeruleus*, *Echis carinatus* and *Naja naja*. Though the ASV is effective in saving lives, its use has many limitations. It is administered to envenomed patients with extreme caution, only when initial evidence of neurotoxicity manifesting as ptosis of the eyes is observed. By that time, the venom would have spread systemically, causing a number of hemostatic changes as mentioned above. One of the manifestations of the hemostatic abnormalities is the hemorrhage (Suvilesh et al., 2017) that occurs at the bite site. The ASV is also known to cause a number of adverse reactions including itching, urticaria, dry cough, nausea, vomiting, diarrhea, tachycardia, fever, etc. (Ahmed et al., 2008; Deshpande et al., 2013; De Silva, Ryan and De Silva, 2016). In addition, it does not address the local effects of the venom such as bleeding, edema, pain etc. (Rucavado, Escalante and Gutiérrez, 2004; Girish and Kemparaju, 2005). Agricultural labourers in India, who are the common targets of N.N bites have poor access to ASV and can ill-afford this expensive treatment. It is well documented that higher doses of ASV are associated with higher incidences of adverse reactions including anaphylaxis (Chube et al., 2016). Any treatment modality that could reduce the dosage of ASV given to the patient would prove beneficial in not only mitigating the adverse reactions but also reduce the cost of treatment. The present study explores such a possibility of supplementation of low concentrations of ASV with a herbal extract.

The methanolic extract of *Andrographis paniculata* (MAP) has shown a number of beneficial effects in counteracting N.N venom toxicity. MAP from leaves and roots, when studied *in-vitro* against N.N venom has shown neutralizing properties against phosphomonoesterase, phosphodiesterase, acetylcholinesterase, phospholipase A_2_, hyaluronidase, L-amino acid oxidase, ATPase and fibrinolytic activities (Gopi et al*.*, 2011; R S A, P C and Gnaniah, 2014; Sivakumar A, Manikandan A, 2015; Mani, 2015). The extract was capable of reducing edema (R S A, P C and Gnaniah, 2014; Rajesh et al., 2017) and hemorrhage (Alam, 2014) *in-vivo* and also prolonged the survival time in mice (Premendran et al. 2011). In the light of encouraging findings obtained with TEG (Nayak, Ahammad, et al*.*, 2020) and inhibition of venom acetylcholinesterase (AchE) and hyaluronidase (Nayak, Kumar, et al*.*, 2020) by a combination of ASV and MAP, it was thought worthwhile to explore the effects of this combined strategy on secondary hemostasis which involves the extrinsic, intrinsic and common pathways of clotting.

### Materials and methods

#### Ethical clearance.

The present study was conducted after obtaining the clearance from Institutional Ethics Committee (IEC: 320/2017), Kasturba Medical College, Manipal Academy of Higher Education, Manipal, Karnataka, India.

#### Blood Samples

Whole blood samples were collected from healthy volunteers using citrated vacutainers, after obtaining their consent to participate in the study. Plasma was separated and used for immediate estimations of PT, aPTT and TT.

#### Naja naja (N.N) venom

Lyophilized form of N.N venom was procured from K.V Institute, Sagarpalli District, Ballia, Uttar Pradesh (License No. 01/Snake 1/2004–05) and stored at 2–8ºC. Venom stock solution was prepared by dissolving 10 mg in 1 ml of 0.08 M phosphate buffer (pH 7.6), stored at 2–8ºC until further use. Aliquots of working venom solutions were prepared using the same buffer. It was observed that N.N venom at 3 µg, caused a measurable change in aPTT and so this concentration was selected for further use. In the case of PT and TT, a higher concentration was required to enable the appearance of a clot and so these experiments were carried out using 10 µg N.N venom.

#### Anti-snake venom (ASV)

Polyvalent ASV was obtained from Bharat Serums and Vaccines Pvt. Ltd, Ambarnath, Maharashtra, India. Contents of the entire vial were reconstituted using 10 ml sterile water provided by the manufacturer and stored at 2–8ºC. According to the manufacturer, 1 ml of reconstituted ASV could neutralize 0.6 mg of N.N venom and contained 2.2 mg protein by Lowry’s method (Randall and Lewis, 1951). For the estimation of PT, aPTT and TT, aliquots of reconstituted ASV were used in the range of 5 µl–17 µl containing 11 µg–37.4 µg protein by Lowry’s method.

#### Methanolic extract of Andrographis paniculata (MAP)

The MAP was sourced from Natural Remedies Pvt Ltd, Bangalore, Karnataka, India (Batch No. BKCEX/2015Lot010) and stored at 2–8ºC. HPLC and ICP-MS analysis of MAP revealed the presence of andrographolide (31.4%) and heavy metals such as lead, cadmium, arsenic and mercury in the extract respectively. GC–MS analysis of the methanolic extract showed the presence of 44 compounds whose MS spectra were matched with NIST library for the confirmation of the compounds (Nayak, Ahammad, et al*.*, 2020). Qualitative analysis of MAP revealed the presence of flavonoids, phenols, tannins, terpenoids, carbohydrates and absence of alkaloids. Quantitative analysis of MAP demonstrated the total phenolic content in the extract as 43.55 mg GAE/g and total flavonoid content as 11 mg QE/g of extract (Nayak, Kumar, et al*.*, 2020).

For the estimation of PT and TT, stock solutions with varying concentrations of MAP were prepared by dissolving 1 mg, 10 mg and 20 mg in 1 ml dimethyl sulfoxide (DMSO-99% pure). Aliquots from the stock solutions containing 10 µg, 100 µg and 200 µg, respectively, were used in the assays.

For the estimation of aPTT, set of 5 stock solutions of MAP containing 3 mg, 6 mg, 12 mg, 15 mg and 18 mg in 1 ml DMSO were prepared and aliquots containing 30 µg, 60 µg, 120 µg, 150 µg and 180 µg were used for the assays based on a pilot study of their effectiveness.

### Statistical analysis

Data was analyzed using SPSS software version 16.0 and comparison of results between the groups was by one-way ANOVA followed by Tukey’s post hoc test. *P* < 0.05 was considered as statistically significant. Results were represented in terms of Mean ± SEM for triplicate samples.

### Methodology

Estimation of aPTT, PT and TT was using commercially available kits.

#### Estimation of aPTT

The aPTT was estimated using CoagTHREE kit from AGAPPE (Bain, 2017). For plasma control, citrated plasma (0.1 ml) from healthy volunteers was incubated with 0.1 ml of aPTT reagent and incubated for 3 min at 37℃. This was followed by the addition of 0.1 ml of pre-warmed 0.02 M CaCl_2_ and clotting time was recorded using a timer.

#### Estimation of PT

The PT was estimated using CoagTHREE kit from AGAPPE (Bain, 2017). Citrated plasma (0.1 ml) from healthy volunteers was pre-warmed at 37℃ for 3 min and 0.2 ml of pre-warmed PT reagent was added (plasma control). Clotting time was recorded using a timer.

#### Estimation of TT

The thrombin time was estimated using TriniCLOT kit from TCoag (Adcock, Hoefner, Kottke-Marchant, Marlar, 2008). Citrated plasma (0.2 ml) collected from healthy volunteers was pre-warmed for 2 min at 37℃ after which 0.1 ml of thrombin reagent was added and time for clot formation was estimated.

All the 3 parameters were assayed in 2 groups of experiments.

**Fig. 1:**
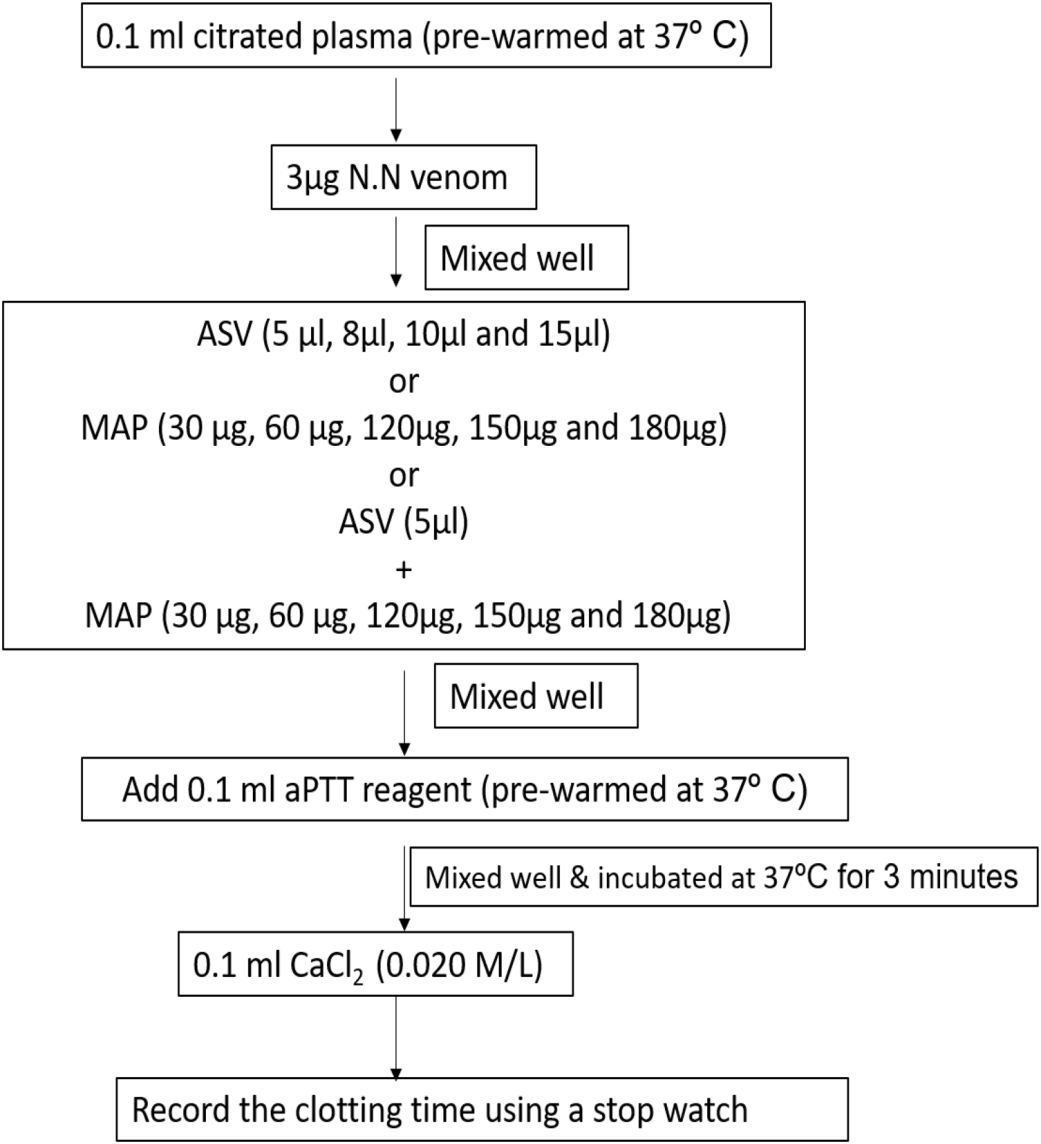
Effects of ASV, MAP and ASV + MAP on N.N venom-induced changes in aPTT without prior incubation of normal citrated plasma with venom. *ASV* anti-snake venom; *MAP *methanolic extract of *Andrographis paniculata*; *N.N* *Naja naja*; *aPTT* activated partial thromboplastin time

**Fig. 2:**
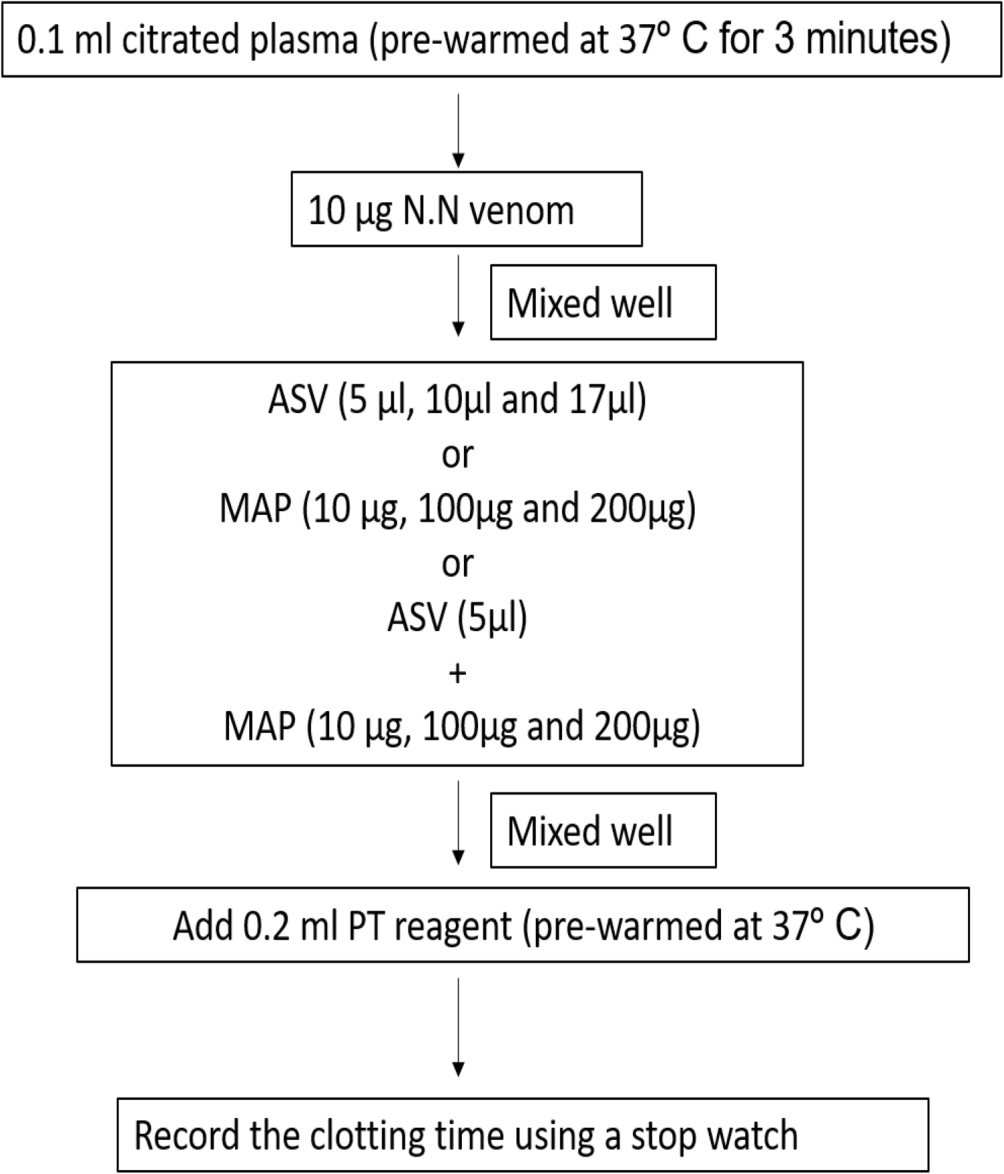
Effects of ASV, MAP and ASV + MAP on N.N venom-induced changes in PT without prior incubation of normal citrated plasma with venom. *ASV* anti-snake venom; *MAP *methanolic extract of *Andrographis paniculata*; *N.N* *Naja naja*; *PT* prothrombin time

**Fig. 3:**
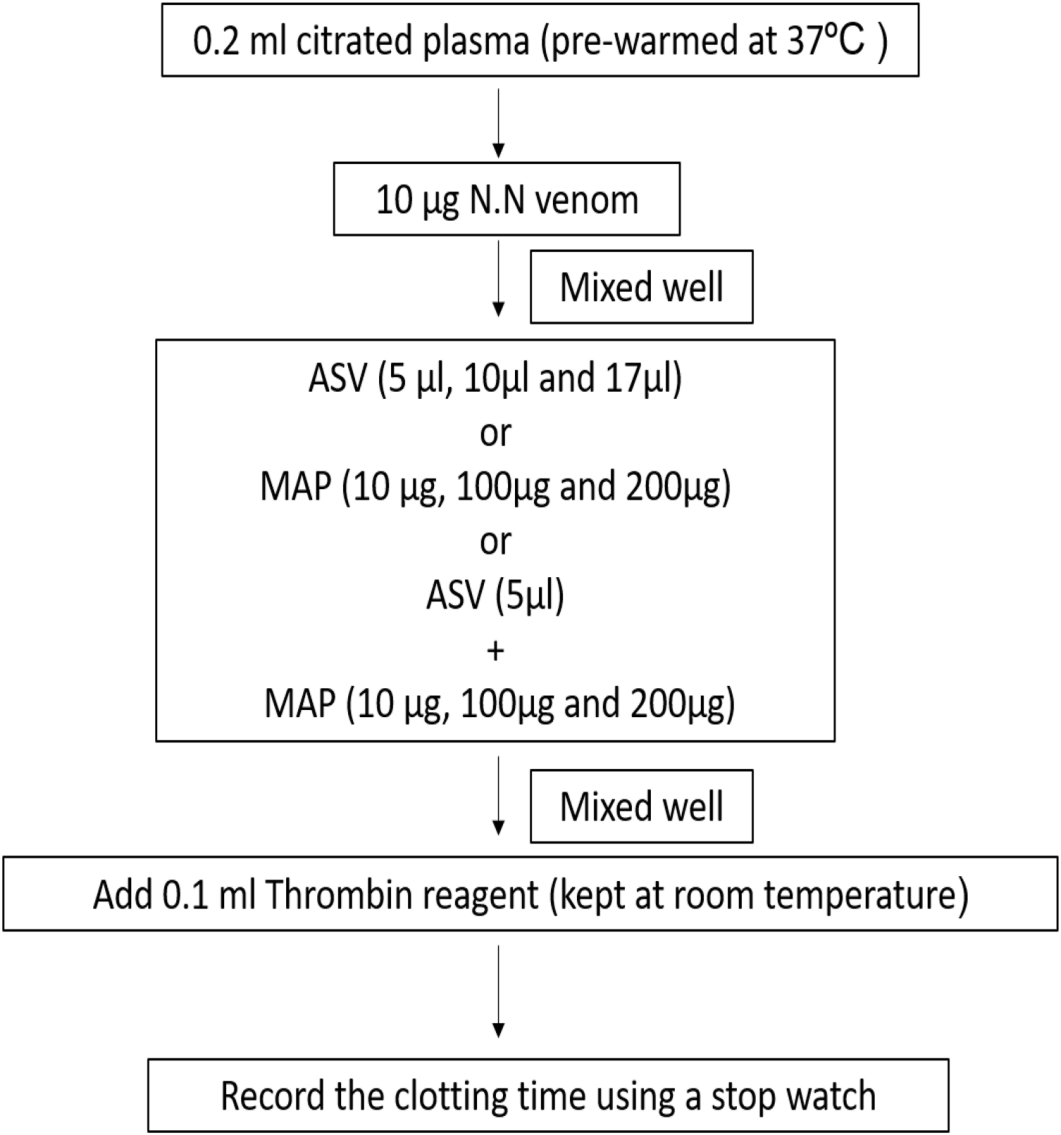
Effects of ASV, MAP and ASV + MAP on N.N venom-induced changes in TT without prior incubation of normal citrated plasma with venom. *ASV* anti-snake venom; *MAP* methanolic extract of *Andrographis paniculata*; *N.N* *Naja naja*; *TT* thrombin time

In **Group I experiments**, the aPTT (Fig. 1), PT (Fig. 2) and TT (Fig. 3) were estimated without prior incubation of the plasma with venom. This set of experiments represent the immediate effect of ASV, MAP or their combination on the intrinsic (aPTT), extrinsic (PT) and common pathways (TT) of clotting.

**Fig. 4:**
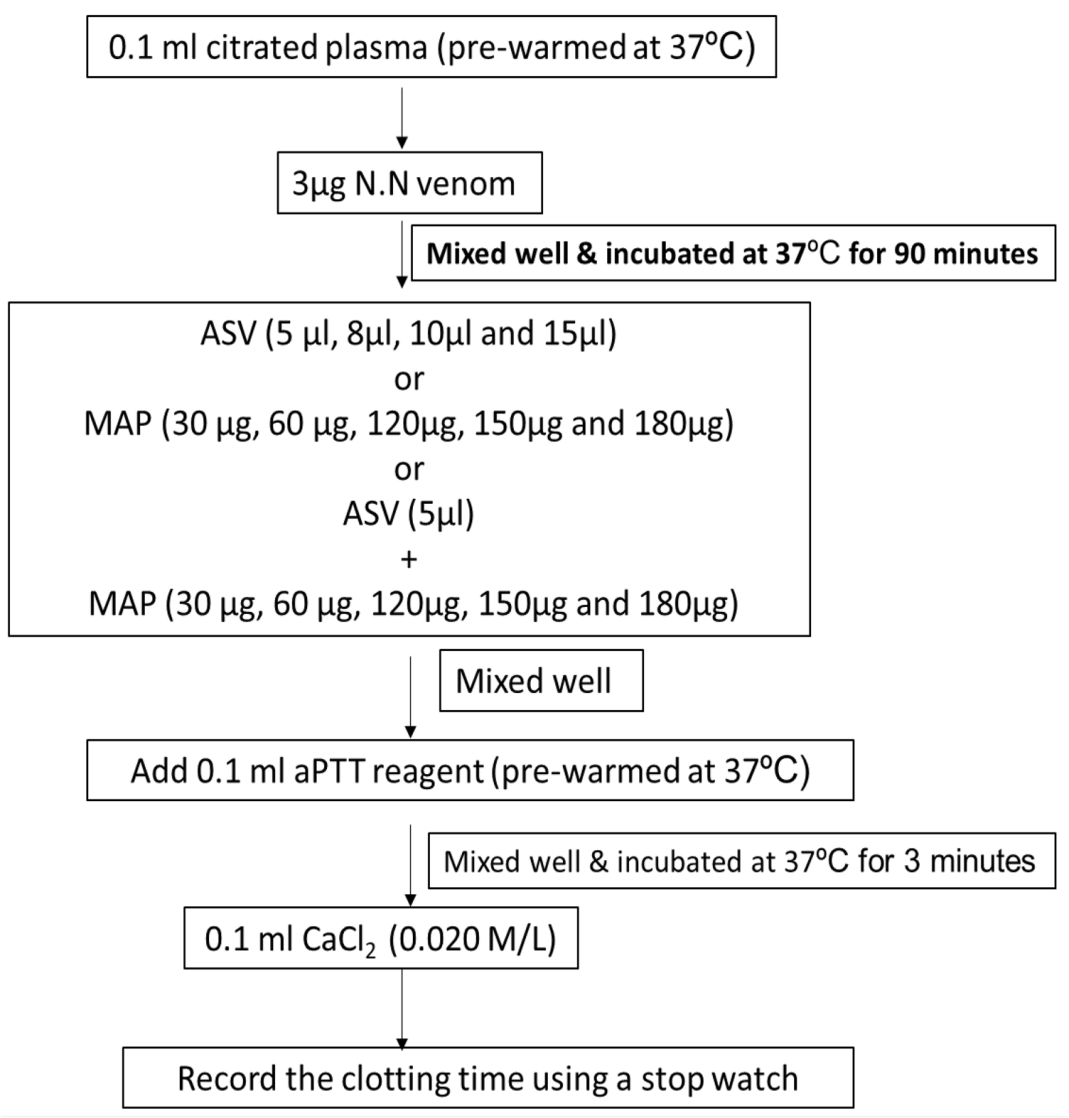
Effects of ASV, MAP and ASV + MAP on N.N venom-induced changes in aPTT with prior incubation of normal citrated plasma with venom at 37ºC for 90 min. *ASV* anti-snake venom; *MAP* methanolic extract of *Andrographis paniculata*; *N.N* *Naja naja*; *aPTT* activated partial thromboplastin time

**Fig. 5:**
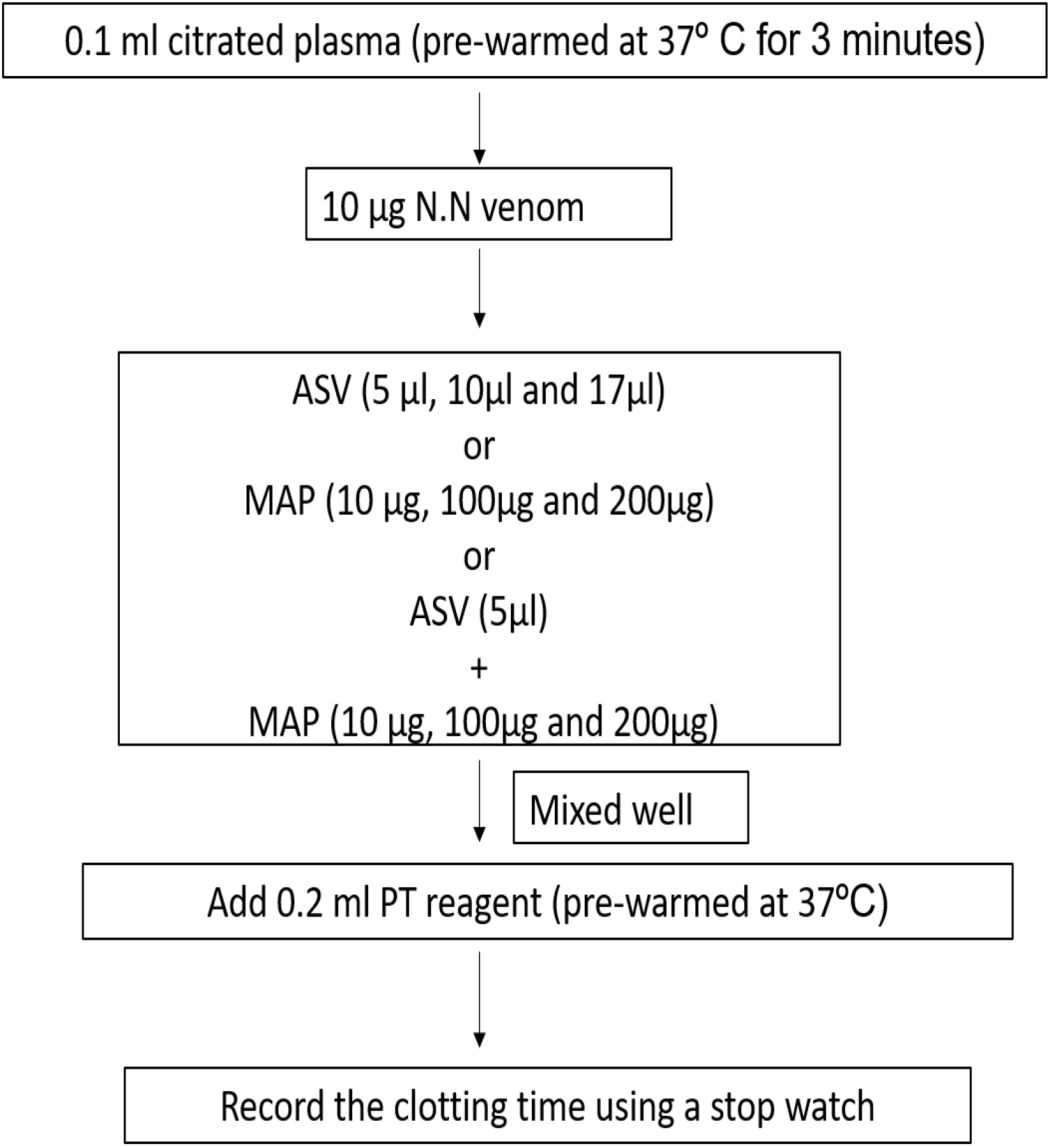
Effect of ASV, MAP and ASV + MAP on N.N venom-induced changes in PT with prior incubation of normal citrated plasma with venom at 37ºC for 90 min.*ASV* anti-snake venom; *MAP* methanolic extract of *Andrographis paniculata*; *N.N* *Naja naja*; *PT* prothrombin time

**Fig. 6:**
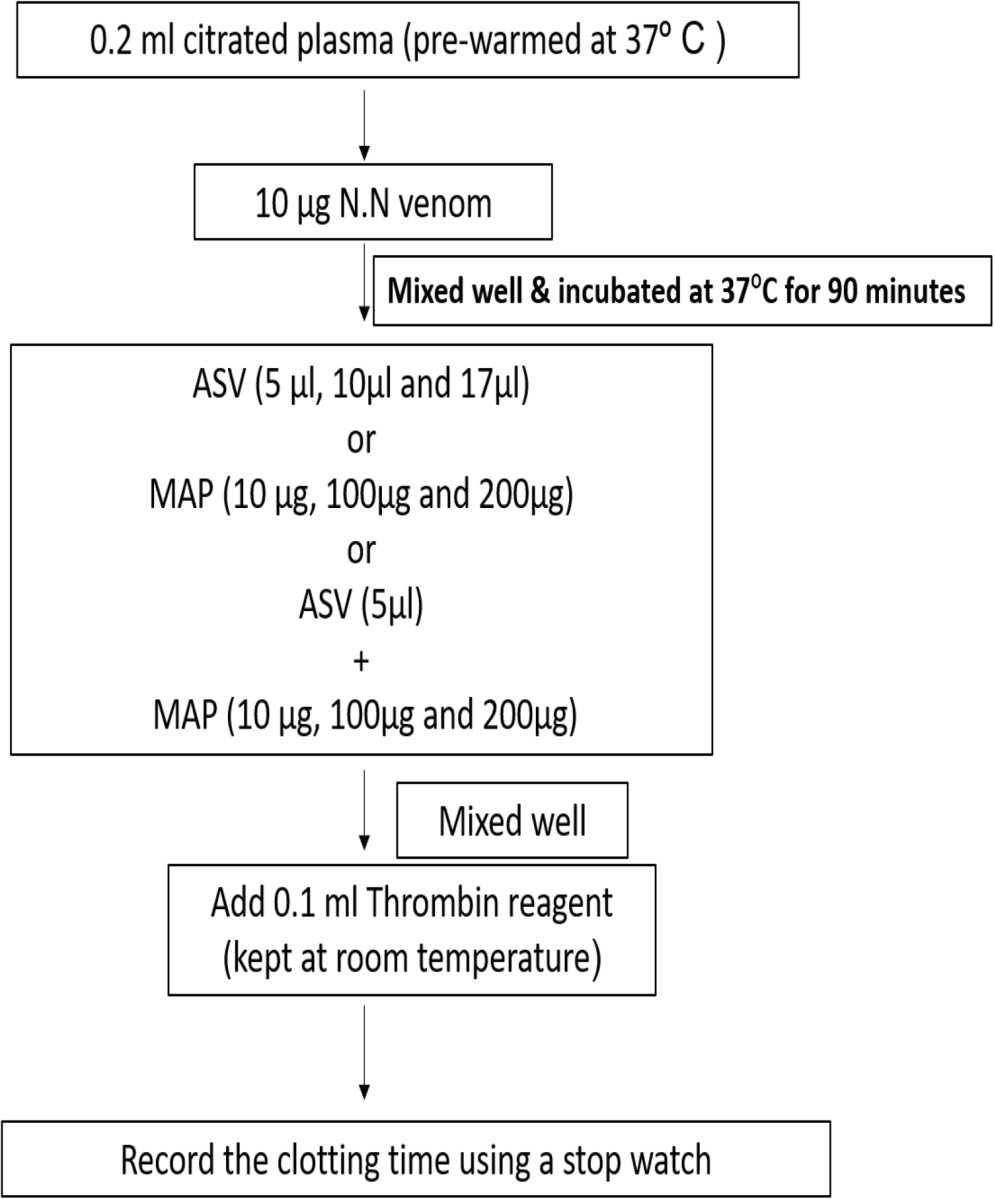
Effect of ASV, MAP and ASV + MAP on N.N venom-induced changes in TT with prior incubation of normal citrated plasma with venom at 37ºC for 90 min. *ASV* anti-snake venom; *MAP* methanolic extract of *Andrographis paniculata*; *N.N* *Naja naja*; *TT* thrombin time

In Group II experiments, the plasma was incubated with venom at 37℃ for 90 min following which the aPTT (Fig. 4), PT (Fig. 5), and TT (Fig. 6) were estimated. These experiments represent the effect of ASV, MAP or their combination in reversing the changes in clotting caused by venom constituents over a period of 90 min. These *in-vitro* experiments simulate the effect the venom on the clotting cascade in a snake bite victim who is brought to the hospital after a period of 90 min following the bite. A time lapse of 90 min was selected because in most victims of N.N bite, delay of more than 90 min causes death due to respiratory paralysis precluding the possibility of any treatment.

### Results

#### Group I experiments

##### Effect on aPTT

**Fig. 7:**
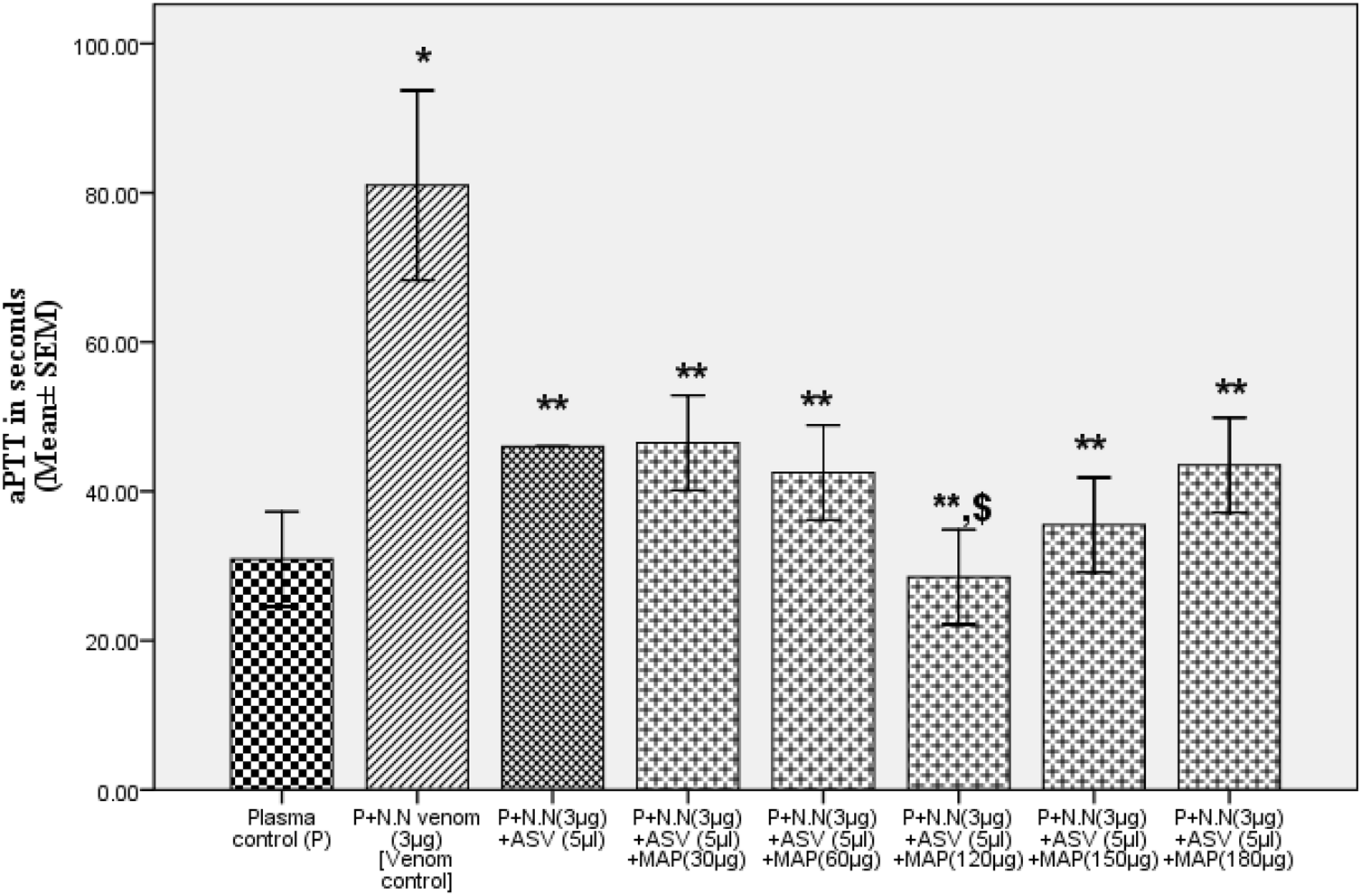
Comparison of effects of combination of ASV and MAP with ASV alone in reducing the N.N venom-induced increase in aPTT without prior incubation of plasma with venom at 37ºC for 90 min. Legends: All values represents mean ± SEM of three values, where *p < 0.05 compared to plasma control (P); **p < 0.05 compared to P + N.N venom (3 µg); ^$^*p* < 0.05 compared to P + N.N venom (3 µg) + ASV (5 µl). *ASV* polyvalent anti-snake venom; *MAP* methanolic extract of *Andrographis paniculata*; *N.N* *Naja naja*; *aPTT* activated partial thromboplastin time; P plasma

Addition of MAP (30-150 µg) to normal citrated plasma, had no effect on aPTT (normal range: 21–38 s as per CoagTHREE kit). The aPTT of normal citrated plasma was prolonged by 3 µg of N.N venom by 175% under standard assay conditions (Table 1). The ASV in the range of 5 µl to 15 µl (11–33 µg in terms of Lowry protein) showed a concentration-dependent effect and was able to bring the aPTT back to normal at 15 µl. The MAP also showed a concentration-dependent effect in the range from 30 to 180 µg in bringing the aPTT towards the normal range. When the ASV concentration was minimized to 5 µl and supplemented with MAP, aPTT was brought back to normal at MAP concentration of 120 µg (Fig. 7). Further increase in MAP concentration up to 180 µg caused a prolongation of aPTT.

**Table 1:** Effects of ASV, MAP and their combination on N.N venom induced changes in aPTT without prior incubation of the plasma with venom

Serial No	Groups	aPTT in seconds (Mean ± SEM)
1	Plasma control (P)	30.9 ± 0.5
2	P + MAP (30 µg) [MAP control-1]	30 ± 0.5
3	P + MAP (60 µg) [MAP control-2]	32 ± 0.1
4	P + MAP (120 µg) [MAP control-3]	32.1 ± 0.2
5	P + MAP (150 µg) [MAP control-4]	35 ± 0.5
6	P + N.N venom (3 µg) [Venom control]	85 ± 3.0 *(*P* = 0.001)
7	P + N.N venom (3 µg) + ASV (5 µl)	46 ± 0 **(*p* = 0.001)
8	P + N.N venom (3 µg) + ASV (8 µl)	43.5 ± 0.5 **(*p* = 0.001)
9	P + N.N venom (3 µg) + ASV(10 µl)	40.1 ± 0.1 **(*p* = 0.001)
10	P + N.N venom (3 µg) + ASV(15 µl)	27 ± 2.0 **(*p* = 0.001)
11	P + N.N venom (3 µg) + MAP (30 µg)	77 ± 7.0
12	P + N.N venom (3 µg) + MAP (60 µg)	69 ± 4.0
13	P + N.N venom (3 µg) + MAP (120 µg)	54 ± 2.0 **(*P* = 0.008)
14	P + N.N venom (3 µg) + MAP (150 µg)	46 ± 4.0 **(*p* = 0.001)
15	P + N.N venom (3 µg) + MAP (180 µg)	46.5 ± 0.5 **(*p* = 0.001)
16	P + N.N venom (3 µg) + ASV(5 µl) + MAP (30 µg)	46.5 ± 0.5 **(*p* = 0.001)
17	P + N.N venom (3 µg) + ASV(5 µl) + MAP (60 µg)	42.5 ± 0.5 **(*p* = 0.002)
18	P + N.N venom (3 µg) + ASV(5 µl) + MAP (120 µg)	29 ± 2.0 **^,$^(*P* = 0.007)
19	P + N.N venom (3 µg) + ASV(5 µl) + MAP (150 µg)	35.5 ± 0.5 **(*p* = 0.001)
20	P + N.N venom (3 µg) + ASV(5 µl) + MAP (180 µg)	43 ± 1.0 **(*p* = 0.001)

All values represent mean ± SEM of triplicate samples where, *p < 0.05 compared to plasma control, **p < 0.05 compared to P + N.N venom (3 µg) [Venom control], ^$^*p* < 0.05 compared to P + N.N venom (10 µg) + ASV (5 µl); 5 µl ASV contain 11 µg Lowry protein

*ASV* polyvalent anti-snake venom, *MAP* methanolic extract of *Andrographis paniculata*, *N.N* *Naja naja*, *aPTT* activated partial thromboplastin time, *P* plasma

##### Effect on PT

**Fig. 8:**
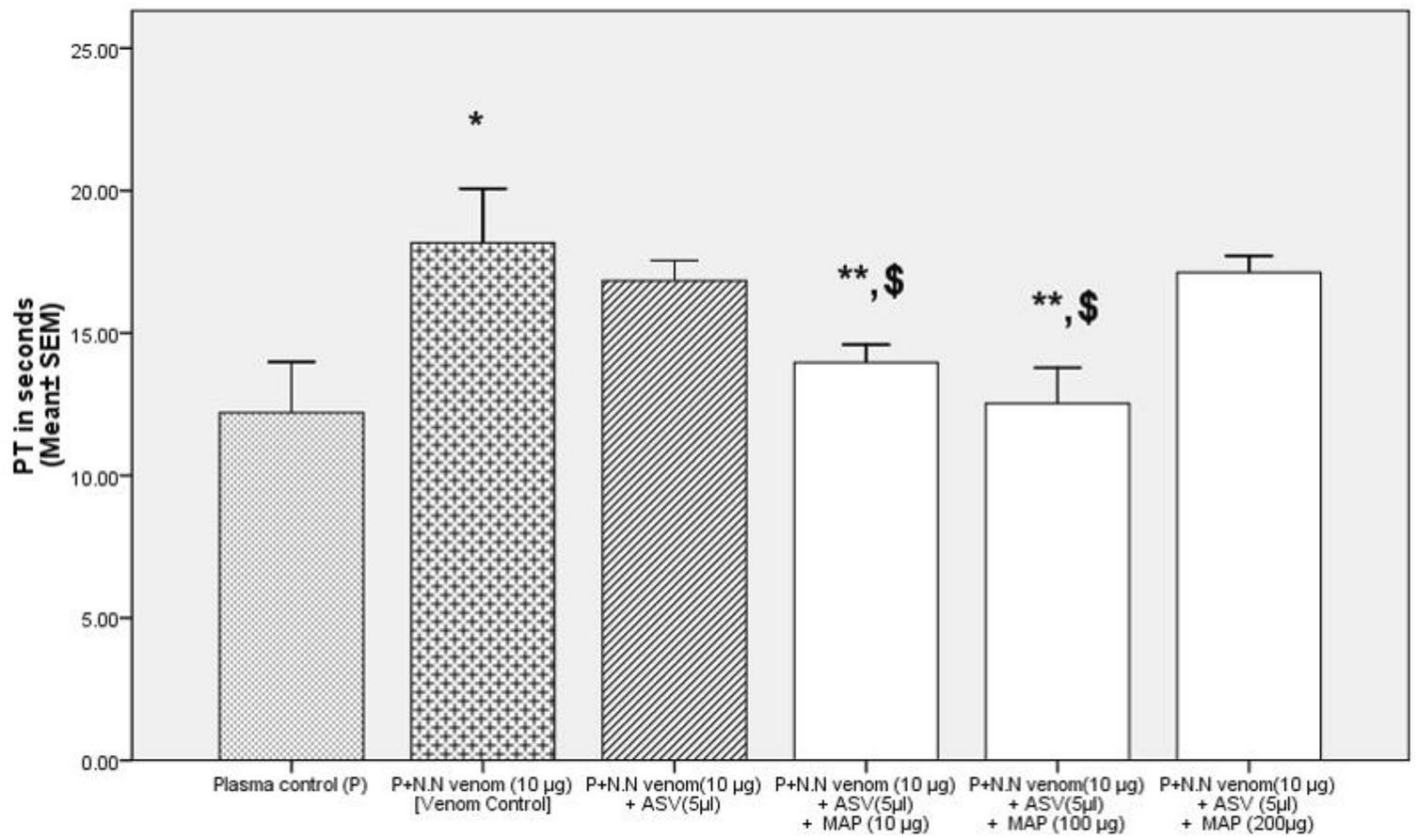
Comparison of effects of combination of polyvalent ASV and MAP with ASV alone on N.N venom-induced increase in PT without prior incubation of plasma with N.N venom. Legends: All values represents mean ± SEM of three values, where *p < 0.05 compared to plasma control; ^**^p < 0.05 compared to P + N.N venom (10 µg) [Venom control]; ^**$**^*p* < 0.05 compared to P + N.N venom (10 µg) + ASV (5 µl). *ASV* polyvalent anti-snake venom, *MAP* methanolic extract of *Andrographis paniculata*, *N.N* *Naja naja*, *PT* prothrombin time; *P* plasma

Addition of MAP (10–200 µg) to normal citrated plasma, had no effect on PT (normal range: 10–15 s as per CoagThree kit). N.N venom (10 µg) was able to prolong the PT by 49% (Table 2). Addition of ASV (5 to 17 µl) to a mixture of plasma and venom, showed a concentration-dependent effect in lowering the PT with a complete reversal to normal level at 10 and 17 µl. MAP also showed a concentration-dependent reversal of PT and brought the PT back to normal at 100 µg. Further increase of MAP to 200 µg prolonged the PT beyond the normal level. When ASV was reduced to 5 µl and supplemented with MAP up to a concentration of 100 µg, the effect was better (26%) than when ASV was used alone (Fig. 8).

**Table 2:** Effects of ASV, MAP and their combination on N.N venom induced changes in PT and TT without prior incubation of the plasma with venom

Serial No	Group	PT in seconds (Mean ± SEM)	TT in seconds (Mean ± SEM)
1	Plasma control (P)	12.2 ± 0.41	14.06 ± 0.06
2	P + MAP (10 µg) [MAP control-1]	13.1 ± 0.3	32 ± 0.5
3	P + MAP (100 µg) [MAP control-2]	14 ± 0.2	36 ± 0.1
4	P + MAP (200 µg) [MAP control-3]	12.5 ± 0.12	38 ± 0.2
5	P + N.N venom (10 µg) [Venom control]	18.16 ± 0.44 *(*p* = 0.001)	18.86 ± 0.27 *(*p* = 0.001)
6	P + N.N venom (10 µg) + ASV (5 µl)	16.8 ± 0.16	17.3 ± 0.42
7	P + N.N venom (10 µg) + ASV(10 µl)	14.5 ± 0.28 **(*p* = 0.001)	16.2 ± 0.2 **(*p* = 0.02)
8	P + N.N venom (10 µg) + ASV(17 µl)	12.16 ± 0.72 **(*p* = 0.001)	13.63 ± 0.18 **(*p* = 0.001)
9	P + N.N venom (10 µg) + MAP(10 µg)	16.5 ± 0.28	41.33 ± 0.88 **(0.001)
10	P + N.N venom (10 µg) + MAP (100 µg)	13.26 ± 0.37** (*p* = 0.001)	22.83 ± 0.6** (*p* = 0.001)
11	P + N.N venom (10 µg) + MAP (200 µg)	17.67 ± 0.16	23.13 ± 0.59** (p = 0.001)
12	P + N.N venom (10 µg) + ASV(5 µl) + MAP(10 µg)	13.9 ± 0.14 **$(p = 0.001)	38.63 ± 0.4 **^,$^(*p* = 0.001)
13	P + N.N venom (10 µg) + ASV(5 µl) + MAP (100 µg)	12.5 ± 0.29 **$(*p* = 0.001)	27.13 ± 0.5 **^,$^(*p* = 0.001)
14	P + N.N venom (10 µg) + ASV(5 µl) + MAP (200 µg)	17.13 ± 0.13	27.7 ± 0.6 **^,$^(*p* = 0.001)

All values represent mean ± SEM of triplicate samples where, *p < 0.05 compared to plasma control (49% increase in PT, 34% increase in TT), ^**^p < 0.05 compared to P + N.N venom (10 µg) [Venom control], ^$^*p* < 0.05 compared to P + N.N venom (10 µg) + ASV (5 µl); 5 µl ASV contain 11 µg Lowry protein

*ASV* polyvalent anti-snake venom, *MAP* methanolic extract of *Andrographis paniculata*, *N.N* *Naja naja*, *PT* prothrombin time, *TT* thrombin time, *P* plasma

##### Effect on TT

Addition of MAP (10–200 µg) to normal citrated plasma prolonged the TT by 128–171%. Addition of 10 µg of N.N venom to citrated plasma (normal range: 10–15 s as per TriniCLOT kit) prolonged the TT by 34% (Table 2). This alteration of TT was completely reversed by the ASV at 17 µl. Addition of MAP (10–200 µg) to normal citrated plasma caused prolongation of TT. Also, in the presence of venom and MAP (10 to 200 µg) the TT was prolonged beyond the normal range. A combination of ASV and MAP was not able to bring TT back to the normal range.

#### Group 2 experiments

##### Effect on aPTT

**Fig. 9:**
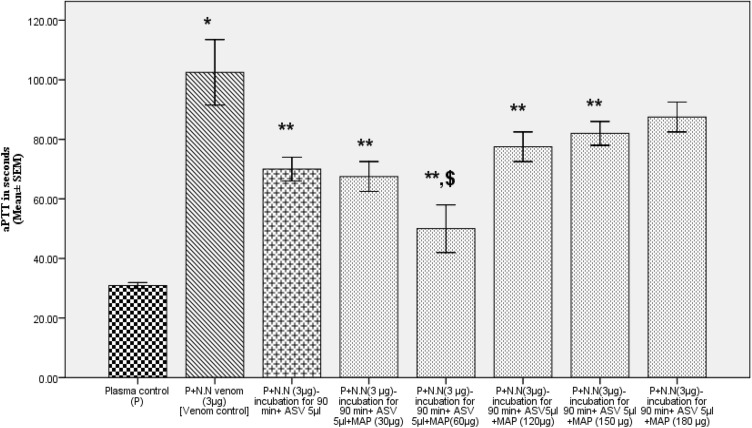
Comparison of effects of combination of ASV and MAP with ASV alone in reducing the N.N venom-induced increase in aPTT with prior incubation of plasma with venom at 37ºC for 90 min. All values represents mean ± SEM of three values, where *p < 0.05 compared to plasma control (231% increase); **p < 0.05 compared to P + N.N venom (3 µg); ^$^*p* < 0.05 compared to P + N.N venom (3 µg) + ASV (5 µl). *ASV* polyvalent anti-snake venom, *MAP* methanolic extract of *Andrographis paniculata*, *N.N* *Naja naja*, *aPTT* activated partial thromboplastin time, P plasma

The aPTT was prolonged to a greater extent by 231% (Table 3) when plasma was incubated with the venom for 90 min prior to the estimation of aPTT. This was 56% more in comparison to aPTT in Group I experiments. Addition of ASV 5 to 8 µl had a limited effect and reduced the prolonged aPTT by 32 to 52%. Further increase in ASV concentration from 10 to 15 µl, prolonged the aPTT, though the aPTT was still shorter by 31–42% when compared to venom control. MAP was also only partially effective (24–45%) in reversing the prolonged aPTT at a concentration of 30 to 60 µg. Further increase in MAP concentration up to 180 µg prolonged the aPTT. Reducing ASV to 5 µl and supplementing it with MAP was maximally effective at a MAP concentration of 60 µg (Fig. 9), showing a 52% reduction in aPTT.

**Table 3:** Effects of ASV, MAP and their combination on N.N venom induced changes in aPTT with prior incubation of the plasma with venom at 37°C for 90 min

Serial No	Group	APTT in seconds (Mean ± SEM)
1	Plasma control (P)	30.9 ± 0.5
2	P + N.N venom (3 µg) [Venom control]	102.5 ± 5.5 *(*p* = 0.001)
3	P + N.N venom (3 µg) + ASV (5 µl)	70 ± 2.0 **(*p* = 0.001)
4	P + N.N venom (3 µg) + ASV (8 µl)	48.5 ± 1.5 **(*p* = 0.001)
5	P + N.N venom (3 µg) + ASV(10 µl)	59 ± 1.0 **(*p* = 0.001)
6	P + N.N venom (3 µg) + ASV(15 µl)	71 ± 1.0 **(*p* = 0.001)
7	P + N.N venom (3 µg) + MAP (30 µg)	78 ± 8.0 **(*p* = 0.05)
8	P + N.N venom (3 µg) + MAP (60 µg)	56.5 ± 2.05 **(*p* = 0.003)
9	P + N.N venom (3 µg) + MAP (120 µg)	75 ± 5.0 **(*p* = 0.03)
10	P + N.N venom (3 µg) + MAP (150 µg)	85 ± 5.0
11	P + N.N venom (3 µg) + MAP (180 µg)	96 ± 4.0
12	P + N.N venom (3 µg) + ASV(5 µl) + MAP (30 µg)	67.5 ± 2.5 **(*p* = 0.001)
13	P + N.N venom (3 µg) + ASV(5 µl) + MAP (60 µg)	50 ± 4.0 **^,$^(*p* = 0.001)
14	P + N.N venom (3 µg) + ASV(5 µl) + MAP (120 µg)	77.5 ± 2.5 **(*p* = 0.007)
15	P + N.N venom (3 µg) + ASV(5 µl) + MAP (150 µg)	82 ± 2.0 **(*p* = 0.018)
16	P + N.N venom (3 µg) + ASV(5 µl) + MAP (180 µg)	87.5 ± 2.5

All values represent mean ± SEM of triplicate samples where *p < 0.05 compared to plasma control (231% increase), **p < 0.05 compared to P + N.N venom (3 µg) [Venom Control]; ^$^*p* < 0.05 compared to P + N.N venom (10 µg) + ASV (5 µl), 5 µl ASV contain 11 µg Lowry protein

*ASV* polyvalent anti-snake venom, *MAP* methanolic extract of *Andrographis paniculata*, *N.N* *Naja naja*, *aPTT* activated partial thromboplastin time, *P* plasma

##### Effect on PT

**Fig.10:**
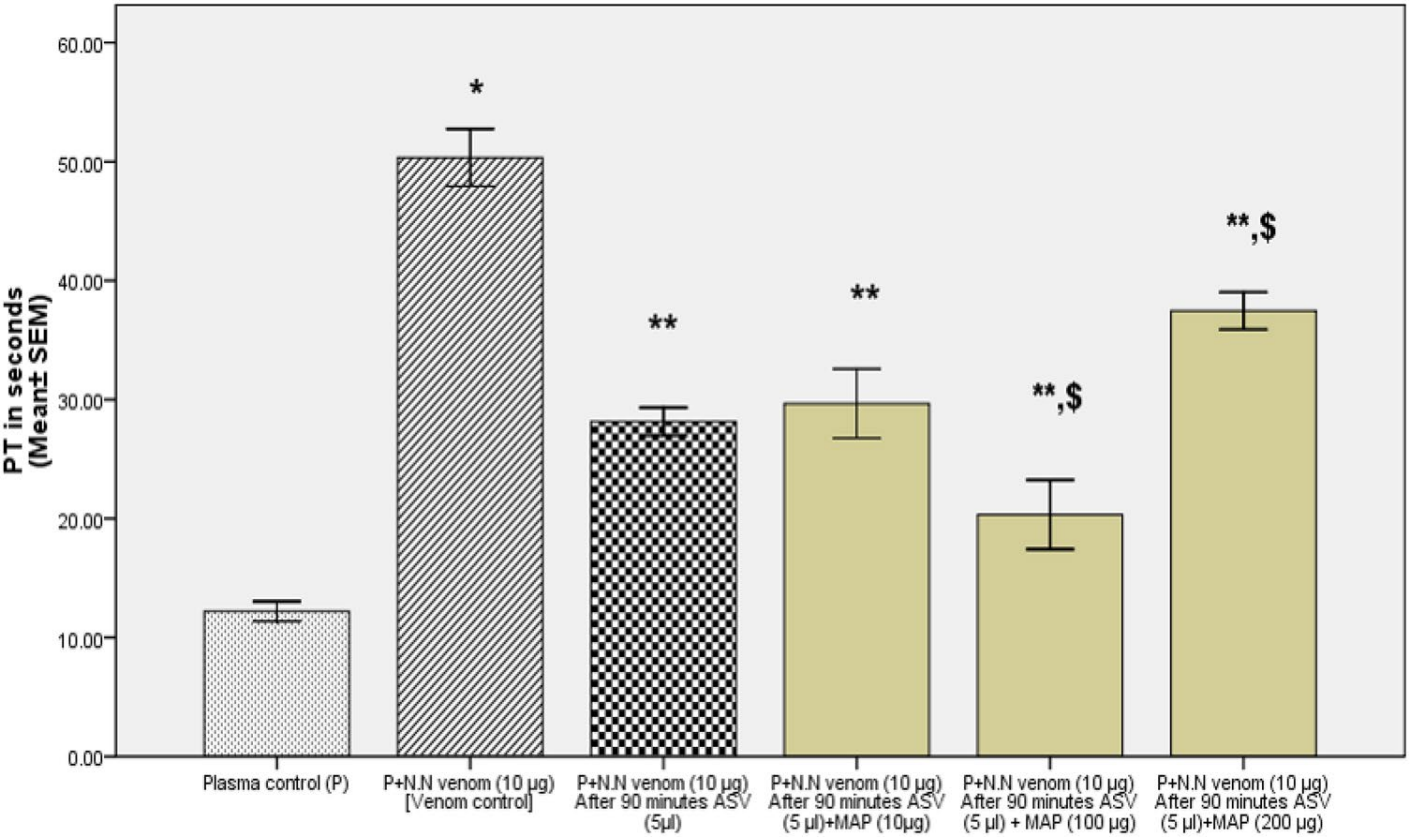
Comparison of effects of combination of polyvalent ASV and MAP with ASV alone on N.N venom-induced increase in PT, with prior incubation of plasma with N.N venom at 37ºC for 90 min. All values represents mean ± SEM of three values, where *p < 0.05 compared to plasma control; ^**^p < 0.05 compared to P + N.N venom (10 µg) [Venom control]; ^$^*p* < 0.05 compared to P + N.N venom (10 µg)—after 90 min ASV (5 µl). *ASV* polyvalent anti-snake venom, *MAP* methanolic extract of *Andrographis paniculata*, *N.N* *Naja naja*, *PT* prothrombin time

Incubation of the plasma with venom for 90 min at 37℃, followed by estimation of PT, caused a drastic prolongation of PT to the extent of 312% (Table 4). ASV was effective in reversing the changes in PT, bringing the PT back to normal at 17 µl. Effect of MAP (10 to 200 µg) on the reversal of PT was marginal (only 17%). When ASV was reduced to 5 µl and supplemented with MAP from 10 to 200 µg, the best effect on PT was observed at ASV 5 µl and MAP 100 µg (Fig. 10). Increasing MAP concentration to 200 µg did not promote a further decrease in PT.

**Table 4:** Effects of ASV, MAP and their combination on N.N venom induced changes in PT and TT with prior incubation of the plasma with venom at 37º C for 90 min

Serial No	Group	PT in seconds (Mean ± SEM)	TT in seconds (Mean ± SEM)
1	Plasma control (P)	12.2 ± 0.41	14.06 ± 0.06
2	P + N.N venom (10 µg) (Venom control)	50.3 ± 1.2 *(*p* = 0.001)	48.46 ± 1.57 *(*p* = 0.001)
3	P + N.N venom (10 µg) + ASV (5 µl)	28.13 ± 0.59 **(*p* = 0.001)	42.8 ± 0.6 **(*p* = 0.002)
4	P + N.N venom (10 µg) + ASV(10 µl)	22.66 ± 0.88 **(*p* = 0.001)	38.3 ± 0.37 **(*p* = 0.001)
5	P + N.N venom (10 µg) + ASV(17 µl)	14.16 ± 0.60 **(*p* = 0.001)	33.6 ± 0.24 **(*p* = 0.001)
6	P + N.N venom (10 µg) + MAP(10 µg)	41.66 ± 0.88 **(*p* = 0.001)	57.2 ± 0.5 **(*p* = 0.001)
7	P + N.N venom (10 µg) + MAP (100 µg)	50.0 ± 1.15	62.8 ± 0.9 **(p = 0.001)
8	P + N.N venom (10 µg) + MAP (200 µg)	54.46 ± 2.26	61.7 ± 0.6 **(*p* = 0.001)
9	P + N.N venom (10 µg) + ASV(5 µl) + MAP(10 µg)	29.66 ± 1.45 **(*p* = 0.001)	48.4 ± 0.8
10	P + N.N venom (10 µg) + ASV(5 µl) + MAP (100 µg)	20.33 ± 1.45 **,$(*p* = 0.001)	52.2 ± 1.02
11	P + N.N venom (10 µg) + ASV(5 µl) + MAP (200 µg)	37.46 ± 1.36 **,$(*p* = 0.001)	60.06 ± 1.2 **,$(*p* = 0.001)

All values represent mean ± SEM of triplicate samples where *p < 0.05 compared to plasma control (312% increase in PT, 245% increase in TT); ^**^p < 0.05 compared to P + N.N venom (10 µg) [Venom control]; ^$^*p* < 0.05 compared to P + N.N venom (10 µg) + ASV (5 µl); 5 µl ASV contain 11 µg Lowry protein

*ASV* polyvalent anti-snake venom, *MAP* methanolic extract of *Andrographis paniculata*, *N.N* *Naja naja*, *PT* prothrombin time, *TT* thrombin time, *P* plasma

##### Effect on TT

N.N venom (10 µg) prolonged the TT significantly by 245% **(**Table 4**)**. ASV had a marginal effect (12–31%) on reversing the TT at a concentration of (5 to 17 µl). MAP prolonged the TT by 18 to 27% (when compared to venom control). When used in combination with ASV (5 µl), MAP showed no significant difference when compared to the use of ASV alone.

### Discussion

As evidenced above, the N.N venom has potent effects on hemostasis. It markedly prolonged the aPTT showing that the intrinsic and common pathways of coagulation involving factor XII, XI, IX, VIII, X, V, II and I is affected. A metalloprotease NN-PF3, from N.N venom with molecular mass 67.81 KDa, has been reported to prolong the aPTT and its activity was inhibited by the Indian polyvalent ASV (Kumar et al., 2010). The present study clearly demonstrated the ability of the polyvalent ASV to bring the aPTT back to normal in a concentration-dependent manner. The MAP also was almost as effective as ASV in reversing the changes in aPTT and effectively worked as a supplement in normalizing aPTT in the presence of low ASV concentrations. Plant extracts used in the treatment of bleeding wounds and snake bite are known to contain compounds such as flavonoids, steroids, terpenoids and alkaloids. These constituents are known to have pro-coagulant property (Liang et al., 2019). Such compounds are also found in MAP (Chen et al., 2020). Quercetin, a compound which is also present in MAP (Veeresham et al., 2013) is known to have hemostatic action (Chen et al., 2020). It is evident that the prior incubation of the plasma with venom caused the irreversible destruction of clotting factors of the intrinsic and common pathway as observed by the prolonged aPTT. Addition of ASV in this condition was relatively less effective and MAP constituents were unable to restore the aPTT to normal. It appears that though they could still bind to venom enzymes, destruction of clotting factors by the venom precluded their action. Similar phenomenon was observed with MAP at higher concentrations of 150 and 180 µg. MAP contains about more than 90 different organic compounds (Sareer, Ahad, Umar 2012) and at higher concentrations, their access to the active sites of enzymes might become limited due to stearic hindrance (Nayak, Ahammad, et al*.*, 2020). Reducing the concentration of ASV to 5 µl and supplementing with MAP (60–120 µg) had consistently a superior effect in Group 1 and Group 2 experiments in reducing aPTT, indicating that the ASV and MAP can supplement each other for a better outcome. It should be noted here that the mechanism by which the venom protease interacts with ASV, is different from that of the interaction of the venom proteases with the MAP constituents. The former depends on antigen–antibody interactions whereas the latter is mainly dependent on the binding to the active sites of venom enzymes as demonstrated by in silico experiments (Gopi et al*.*, 2011; Chayamiti, Mwenje and Mahamadi, 2013).

Prothrombin time is a measure of the functioning of the extrinsic and the final common pathways of the coagulation cascade. This consists of tissue factor and factors VII, I (prothrombin) V X, and fibrinogen. The fact that PT was prolonged by the venom to the extent of 49% and 312% respectively, without and with prior incubation of the venom with plasma, confirms that the venom contains factors that interfere with the extrinsic pathway of coagulation as also observed by others (Kumar et al., 2010). The finding that ASV was able to reverse the change in PT completely even after exposure of the plasma to venom for 90 min, indicates that the venom enzymes interact with the clotting factors without damaging their function, may be by forming complexes which dissociate on the addition of ASV. Kunitz-type proteinase inhibitors with molecular weight of 6.2 KDa and 6.4 KDa have been isolated from N.N venom (Shafqat, Zaidi and Jörnvall, 1990). These are serine proteinase inhibitors and are capable of binding to serine proteinases (Suvilesh et al. 2017) of the clotting cascade such as factor VIIa, Xa, IIa, possibly prolonging PT. NN-PF3, a metalloprotease from NN venom with molecular mass 67.81 KDa, has fibrin(ogen)olytic activity (Kumar et al., 2010) and has been shown to prolong PT but does not prevent clotting. This enzyme cleaves the α-chain of fibrinogen and the reaction has been observed *in-vivo* in mice (Kumar et al., 2010). However, this cleavage happens at a site on fibrinogen without abolishing clotting completely. In addition, N.N venom is known to contain a prothrombin activator which promotes clot formation *in*-*vitro*. This clot is said to have an altered structure with low in elasticity (Sundell et al., 2003). The venom also has a weak plasmin-like activity (Gowtham et al., 2012) which could delay the clot formation by increasing fibrinolysis. Low concentrations of ASV (5 µl) supplemented with MAP (100 µg) was not only highly effective in normalizing PT in both Group 1 and Group 2 experiments, but also had a potentiating effect. Such observations have been made previously by Gomes et al. with *Naja kaouthia* venom and the methanolic extract of *Pluchea indica* (Gomes et al., 2007) and also by Chatterjee et al. with *N. kaouthia* and *Hemidesmus indicus* (Chatterjee, Chakravarty and Gomes, 2006). This gives credence to a combination therapy of using an herbal extract in addition to ASV in N.N bite.

The TT measures the time taken for the conversion of fibrinogen to fibrin by thrombin, a serine protease in the common pathway of clotting. ASV was effective in normalizing TT in Group 1 experiments, which proves that enzymes such as NN-PF3 in N.N venom (Kumar et al., 2010) which degrade fibrinogen and thereby prolong the TT, are neutralized by ASV. However, when the venom was given sufficient time to degrade the fibrinogen in plasma as in Group 2 experiments, the effect of ASV was very limited due to the irreversible nature of the digestion by fibrinogenolytic enzymes.

The coagulation cascade is also known to be inhibited by various phytochemicals such as polyphenols, sulfated polysaccharides through inhibition or decreased activity of thrombin, as well as potentiation of heparin co-factor II (Cordier and Steenkamp, 2012). Ineffectiveness of MAP in reversing TT seems to be due to loss of fibrinogen caused by N.N venom enzymes.

### Conclusion

This study was an evaluation of the ability of ASV, MAP and their combination to normalize the hemostatic abnormalities induced by NN venom. The Indian polyvalent ASV was found to be effective in reversing the deleterious effects of the venom on the intrinsic, extrinsic and common pathways of clotting when the addition of venom was immediately followed by the addition of ASV. When the venom was incubated with plasma prior to the experiments, ASV was partially effective in reversing aPTT and TT, and could completely normalize PT. MAP when used alone, partially improved the aPTT and completely normalized PT. When used along with ASV, the effect of MAP on PT was interesting, in that, it had a potentiating effect on the action of ASV in normalizing it. However, MAP had a deleterious effect on TT, prolonging it beyond control values. However, the beneficial effects of supplementing ASV with MAP have to be assessed comprehensively. In addition to causing hemostatic abnormalities, by virtue of the presence of 81 different toxins, the NN venom affects all organ systems. It has already been conclusively proven that a combination of ASV and MAP are effective in preventing toxic systemic effects such as spreading of the venom by inhibiting hyaluronidase and neurotoxicity by inhibition of venom acetylcholinesterase (Nayak, Kumar, et al*.*, 2020). This study thus conclusively evaluated and proved that the beneficial effects of the combination of ASV with MAP, were multifaceted. The merits extend not only to the mitigation of hemostatic abnormalities but also to a drastic reduction of the use of ASV by 70%. In the Indian context, where the number of snake bites are as many as 50,000 per year, such a strategy implies a better outcome with limited resources, thus reducing the economic burden on snakebite victims.
